# Early mortality after chemotherapy as a quality indicator—the leukemia perspective

**DOI:** 10.1038/s41408-023-00956-x

**Published:** 2023-12-01

**Authors:** Hagop Kantarjian, Mary Alma Welch, Koji Sasaki

**Affiliations:** From the Department of Leukemia, The University of Texas MD Anderson Cancer Center, Houston, USA

**Keywords:** Quality of life, Prognosis

The early mortality rate after a medical procedure or treatment has been a historical measure of quality of care delivery. It was originally developed to assess the risk of surgeries or other procedures and later adopted across medical practices to evaluate differential risks of early mortality with similar therapies to define favorable or unfavorable outliers, and to improve delivery of care. Its subsequent adoption in cancer care had broad applications in areas with widely divergent environments and influencing factors. Some of the variables include the kind of tumor (solid tumor or liquid tumor [leukemia, myeloma, lymphoma]); the type of surgery (extremes of Whipple procedure or lymph node biopsy); the stage (early or advanced); the treatment intensity (intensive chemotherapy, lower-intensity therapy, stem cell transplantation, targeted therapy, immunotherapy); and the risk of mortality with intensive chemotherapy (compromised bone marrow in leukemia or intact bone marrow reserve in solid tumors) [1, 2].

In cancer, the general measure has been the rate of early mortality following some form of treatment, early mortality being defined in some analyses as death within 4 weeks of receiving chemotherapy, as well as receiving chemotherapy within 2 weeks of death in patients with advanced cancers. Because of the broad range of tumors, patients, treatments and environment factors influencing this early mortality, its value as a measure of quality of care has been questioned (Table 1) [3, 4]. Despite this, it has been adopted as a quality index by several “performance improvement” groups and is used to rank healthcare provision across many geographic areas in the US and the world [5, 6].

**Table 1:** Issues with the current quality indicators for early mortality in leukemia.

• Mixing solid tumors with leukemia and other liquid tumors: The latter have significant long-term cure rates in salvage 1 and beyond; hence, the risks of treatment are justifiable, even with the potential for early mortality.
• Mixing frontline and salvage leukemia and other liquid tumors: Highly specialized centers have a larger proportion of salvage vs frontline referrals. This translates into a higher mortality in salvage therapy.
• Specialized leukemia centers offer multiple novel investigational therapies. This results in higher rates of mortality within 4 weeks as patients are receiving such promising therapies.
• Pressuring healthcare providers to discontinue therapy to avoid “chemotherapy in the last 2–4 weeks of life” could be potentially harmful to patients, and may shorten survival and worsen the quality of life as a result of well-known tumor rebound effects
• Intensive chemotherapy in the setting of marrow compromise (leukemia) is expected to result in myelosuppression complications (fever, infections, sepsis, bleeding) that necessitate prompt readmissions and interventions to reduce mortality. Penalizing healthcare facilities for readmissions within 4 weeks of chemotherapy may pressure providers to avoid readmissions, which may increase outpatient mortality.

In leukemia, the mortality rate has been used in two almost diametrically opposed situations, with different interpretations, to measure the quality of care: (1) 4-week mortality after frontline induction chemotherapy in patients with newly diagnosed acute leukemia; and (2) death within 2 (or 4 weeks) weeks of therapy in “terminally ill” (always clear in hindsight) patients with advanced or previously treated leukemia.

In the first situation, death within 4 weeks of induction therapy can reflect a treatment that is too toxic. With similar treatments administered in different locations, the 4-week mortality may delineate relative expertise and supportive care levels. For example, in patients with newly diagnosed acute myeloid leukemia (AML) undergoing standard intensive induction chemotherapy (“3+7,” daunorubicin or idarubicin for 3 days and cytarabine for 7 days), the 4-week mortality rate was 15% in academic centers versus 29% in non-academic centers; it was 12% in NCI-designated cancer centers versus 24% in non-NCI-designated cancer centers [7, 8]. At MD Anderson, the 4-week mortality rate was <5% in younger/fit patients who received intensive induction chemotherapy, and 2% in older/unfit patients treated with lower-intensity induction therapy [9–11].

In the second situation, therapy given “at the end of life” is frequently viewed negatively by supportive care experts and is interpreted to reflect inappropriate care/poor judgment, where the patients might have been unnecessarily harmed by toxic therapy and died in an environment not to their preference. The argument goes that, if well-advised about their treatment options and realistic outcomes, patients might choose to forgo therapy and die at home or in hospice rather than in the hospital. Hence, the delivery of therapy within 2 weeks of death to such patients may negatively impact the quality of care score in a particular environment or hospital.

But the view that withholding therapy in the last 2 weeks of life is a reliable indicator of the quality of care may not consider several important factors: (1) Novel, more effective targeted therapies and immunotherapies with favorable toxicity profiles are now producing startlingly positive results and potential long-term responses in previously incurable patients with leukemia, lymphoma and myeloma. For example, patients who are in second salvage or later can still get significant benefit from treatment with bi-specific T-cell engagers (BiTEs), chimeric antigen receptor T cellular therapy (CART), and other drugs targeting molecular abnormalities [12–17]. (2) Discontinuing targeted therapies when end of life is anticipated to be near may cause tumor rebound effects and worsen the quality of life and shorten life expectancy. (3) Despite physicians’ best efforts to predict outcomes based on patient co-morbidities in order to select patients most likely to benefit from therapy, oftentimes morbidity is related to tumor-associated factors and improves with therapy. Thus, predicting mortality is subjective and unreliable [18–23].

Next, we will discuss in more detail the two opposing situations.

In frontline therapy, healthcare experts agree that the 4-week mortality is a reliable indicator of quality of care but emphasize that it is crucial to consider the population mix. A general hospital with fewer than 20% of patients with cancer and 1% of patients with leukemia will score on average a lower total 4-week mortality than a dedicated cancer hospital. A cancer center hospital with 5% of beds occupied by leukemia patients will score on average a lower 4-week mortality rate than one with 25% of beds with leukemia patients (higher induction mortality in leukemia than in solid tumors due to the more intensive chemotherapy needed and the compromised marrow reserve; many solid tumor induction chemotherapy regimens delivered in the outpatient setting and patients only hospitalized with complications). A cancer hospital that serves as a referral center and offers investigational options will have a higher proportion of patients on salvage versus frontline treatment, which results in a higher 4-week mortality rate since patients on salvage therapy tend to be sicker and more likely to die. Such patients treated at referral centers are often self-selected in that they have the social and financial resources to travel and seek more therapies regardless of the odds. They may often reject the advice of the cancer and supportive care experts who recommend hospice or terminal care. Quality healthcare experts argue that such differences in mortality rates (solid versus leukemia; leukemia frontline versus salvage therapy) would be nullified once all patient-associated co-morbidities were accounted for. However, this did not turn out to be correct because of patient- and tumor-associated latent (unmeasured) variables that are not factored in such analyses [24, 25]. While healthcare performance improvement companies such as Vizient, Premier, Nordic, HeathTrust, Optum, and GHX claim to account for such differences and to “compare apples to apples,” latent variables account for almost two-thirds of the variances in comparative studies [24, 25]. Thus, the best way to account more precisely for differences in the 4-week mortality based on the population mix is to introduce quality measure codes that account for cancer subsets (leukemia, stem cell transplant, solid tumor) and line of therapy (frontline, salvage) with associated hazard ratios/mortality indices that describe the expected mortality rates from existing data. For example, leukemia will have a 2–4 times worse “mortality index” than a solid cancer; a solid cancer or leukemia in later-line therapies will have a twofold worse “mortality index” than frontline chemotherapy [24].

How about therapy given within 2 weeks of death in a terminally ill cancer patient? Here, the distinction between solid tumors and leukemia, and recently other hematologic cancers such as lymphoma and multiple myeloma, is important. In solid cancers, the tumor is already advanced and/or metastatic, the poor prognosis well-defined, and poor quality of life irreversible (cancer-associated pain requiring narcotics; no effective salvage chemotherapy; median survival of less than 3–12 months). Thus, the administration of chemotherapy in the last 2–4 weeks of life may not be of value. Leukemia is a different situation, where, for example, patients with acute lymphoblastic leukemia (ALL) in first or second salvage and beyond may still be rescued with effective combinations of chemotherapy and novel targeted or immune therapies, followed by allogeneic stem cell transplantation or CAR T-cells [12, 13]. The potential long-term survival ranges from 20 to 60% depending on the prior therapies and remission duration. However, such therapies are associated with mortality rates of 5–20+% from complications of myelosuppression (infections, bleeding) or other treatment-related toxicities (e.g., cytokine release syndrome). Hence “receipt of chemotherapy in the last two (or four) weeks of life” is associated with anticipated risks, rather than being a poor-quality indicator. In essence, a good benefit:risk approach. The same applies to salvage patients with lymphoma and myeloma, where today, novel immune strategies such as CAR T-cells and BiTEs are producing unprecedented response rates, durable remissions and potential long-term survival rates of 20% to 50+% [14–17].

An important recent issue is the blurring of the line between active chemotherapy used as a treatment and as a supportive care measure. In solid tumors, the separation is clear: chemotherapy to shrink the tumor; narcotics as supportive care to control tumor-associated pain. In leukemia, pain is rare and usually alleviated with a short course of steroids, but supportive care involves addressing three common components: red cell transfusions to improve anemia and fatigue/poor quality of life; platelet transfusions for thrombocytopenia and bleeding; and antibiotics to prevent or treat infections associated with immunosuppression. But leukemia also has an added dimension not observed in solid tumors: a rise in peripheral blood blasts that compromises organ function, worsens quality of life, causes pain and shortens survival by, at times, several months. This can be ameliorated with agents classified as chemotherapy but that also can be used to control the peripheral blast counts as a supportive measure: hydroxyurea, cytarabine, hypomethylating agents (azacitidine, decitabine). We believe that including such uses in the quality indication if patients receive them within 2 weeks of death may be counterproductive and potentially harmful if treating physicians feel pressured to withhold a measure that might benefit the patient.

With the ongoing therapeutic revolution involving novel small-molecule targeted therapies and immunotherapies (BCR::ABL1, FLT3, IDH, and JAK2 tyrosine kinase inhibitors; CD19/20/22 CAR T-cells and BiTES; others) [20, 21], we have learned new lessons. Three examples are illustrative. In the early days of investigations into BCR::ABL1 TKIs for chronic myeloid leukemia (CML), patients were required by protocol to withhold a prior TKI for 2 weeks to be eligible for the novel TKIs. Some patients who were in the CML chronic phase with evidence of TKI resistance stopped the therapy and quickly progressed to accelerated or blast phases. This led to the standard that any patient with CML ideally should be on a daily TKI dose throughout their disease course, be it in the chronic phase or in transformation (in combinations). In myelofibrosis (MF), withholding a JAK2 inhibitor (ruxolitinib) for even a week can result in severe rebound splenomegaly and worsening MF-associated symptoms (joint pain, sweats, pruritus, weight loss). In chronic lymphocytic leukemia (CLL), withholding a Bruton tyrosine kinase (BTK) inhibitor (during protocol-required transitions or in practice for other reasons) results at times in a massive rebound progression or even Richter’s transformation, and multi-organ failure. These three situations highlight the importance of continuing such targeted therapies in leukemia and other liquid tumors.

In summary, the 4-week mortality is a good quality indicator in frontline acute leukemia therapy. In leukemia salvage, such intensive chemotherapy with or without other novel therapies followed by allogeneic SCT may be curative but results in higher 4-week mortality rates that should be incorporated into the quality indicators. Finally, several therapies classified as “chemotherapy” but sometimes used as supportive care should be allowed without penalty and considered as favorable quality-improving measures even in the last 2–4 weeks of life.
